# Tongue crack recognition using segmentation based deep learning

**DOI:** 10.1038/s41598-022-27210-x

**Published:** 2023-01-10

**Authors:** Jianjun Yan, Jinxing Cai, Zi Xu, Rui Guo, Wei Zhou, Haixia Yan, Zhaoxia Xu, Yiqin Wang

**Affiliations:** 1 Shanghai Key Laboratory of Intelligent Sensing and Detection Technology, East China University of Science and Technology, 130 Meilong Road, Shanghai, 200237 China; 2 Comprehensive Laboratory of Four Diagnostic Methods, Shanghai University of Traditional Chinese Medicine, Shanghai, 201203 China

**Keywords:** Image processing, Machine learning

## Abstract

Tongue cracks refer to fissures with different depth and shapes on the tongue’s surface, which can characterize the pathological characteristics of spleen and stomach. Tongue cracks are of great significance to the objective study of tongue diagnosis. However, tongue cracks are small and complex, existing methods are difficult to extract them effectively. In order to achieve more accurate extraction and identification of tongue crack, this paper proposes to apply a deep learning network based on image segmentation (Segmentation-Based Deep-Learning, SBDL) to extract and identify tongue crack. In addition, we have studied the quantitative description of tongue crack features. Firstly, the pre-processed tongue crack samples were amplified by using adding salt and pepper noise, changing the contrast and horizontal mirroring; secondly, the annotation tool Crack-Tongue was used to label tongue crack; thirdly, the tongue crack extraction model was trained by using SBDL; fourthly, the cracks on the tongue surface were detected and located by the segmentation network, and then the output and features of the segmentation network were put into the decision network for the classification of crack tongue images; finally, the tongue crack segmentation and identification results were quantitatively evaluated. The experimental results showed that the tongue crack extraction and recognition results based on SBDL were better than Mask Region-based Convolutional Neural Network (Mask R-CNN), DeeplabV3+, U-Net, UNet++ and Semantic Segmentation with Adversarial Learning (SegAN). This method effectively solved the inaccurate tongue crack extraction caused by the tongue crack’s color being close to the surrounding tongue coating’s color. This method can achieve better tongue crack extraction and recognition results on a small tongue crack data set and provides a new idea for tongue crack recognition, which is of practical value for tongue diagnosis objectification.

## Introduction

**Figure 1:**
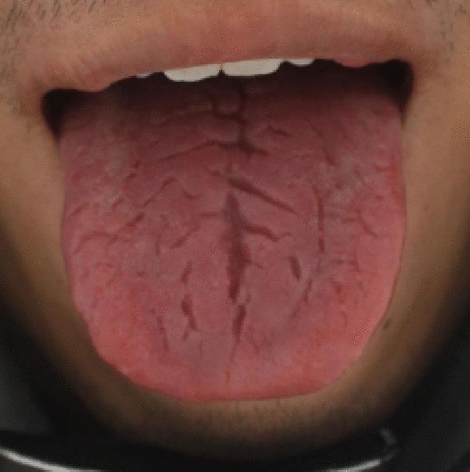
Typical image of cracked tongue.

In recent years, machine vision has been developed with the development of computer software and hardware, and the combination of medical diagnosis and computer vision has gradually become a hot topic. Researchers combined tongue diagnosis with image processing and machine learning in order to achieve the objectification and modernization of tongue diagnosis. This method reduced the interference of environmental factors on the tongue image and avoid doctors’ inaccurate judgment due to subjective factors, thereby achieving the effect of objective tongue diagnosis and improving the accuracy of judgment. At present, researchers have made considerable progress, but there are still some problems that are difficult to overcome. The research on tongue image mainly focuses on the tongue coating and tongue color^1,2^, but there are few studies on the tongue crack, and it is difficult to make breakthrough progress. Tongue cracks can effectively reflect some diseases and can be further diagnosed in combination with other tongue features^3^. As shown in Fig. 1, the tongue crack is small and complex^4^, so it is difficult to extract them effectively.

There are several studies on segmentation and recognition of tongue cracks, and early studies almost concentrated on the methods of threshold segmentation and line detection. The threshold method uses the grayscale difference or color difference between the tongue crack and other parts of the tongue surface to establish a threshold for segmentation, while the line detection method calculates the speed of change in the color or grayscale brightness of the edge of the tongue crack, so as to obtain the contour of the crack. Rhee^5^ used U-Net and an adaptive threshold technique to extract tongue cracks within post-processing. Liu et al.^6^ attempted to extract tongue cracks by using a method based on the wide line detector which extracts the whole of the line by employing an isotropic nonlinear filter. Li et al.^7^ proposed a new method using statistic feature extracted by wide line, such as Max-distance, to train a binary SVM as a classifier for cracked tongue. And now more deep learning methods are used for crack defection based on classification, pixel segmentation and object detection^8–10^. Weng et al.^11^ proposed to train the tooth-mark and crack detection model by using tongue images annotated bounding-box. It was a weakly supervised method that added several classification branches to recognize the tooth-marked tongue and cracked tongue according to the YOLO object detection model. Peng et al.^12^ proposed a *P*-type neural network architecture based on a lightweight encoder-decoder structure which could get the detailed extraction result at pixel level. Xue et al.^13^ proposed to use cracked and non-cracked regions to train Alexnet to extract deep features of cracked regions. This method focuses on localized cracked regions and trains a multi-instance support vector machine (SVM) to make the final decision. However, the effectiveness and generalization of these methods still need to be improved.

The threshold segmentation method only considers the characteristics of a certain pixel, which doesn’t consider the deeper semantic features, so tongue cracks cannot be extracted well by traditional methods. Only obtaining the tongue crack pixel features on the tongue surface cannot describe the tongue crack well, it is necessary to dig deeper semantic features of tongue images. Therefore, accurate segmentation of tongue crack can be achieved by combining the shallow features with the deep features. The convolutional neural network can gradually extract the semantic features of pictures from shallow to deep and use these features as an important basis for classifying cracks and surrounding tongue. It has achieved good results in medical image processing and other fields. However, the color of the crack in the tongue image is similar as the surrounding area, and its main feature is that its shape is a zigzag strip of a certain width. In addition, not only are the crack widths of the same tongue image different, but also the width of the same crack may change as the crack end extend. So it is difficult for semantic segmentation algorithms to deal with the segmentation of the tongue crack. SBDL is a two-level special network with a segmentation network and a decision network, which achieved accurate results for the segmentation of small objects with fewer samples^14^.

Therefore, this paper applies a deep learning network SBDL to extract and identify tongue cracks. The rest of this article consists of five parts. Section "Tongue crack extraction based on SBDL" described the tongue crack extraction based on SBDL. Section "Results" described the details of the experimental and reported the results. The results of the experiment were discussed in Section "Discussion". Finally, the conclusion was drawn in Section "Conclusion".

## Tongue crack extraction based on SBDL

**Figure 2:**
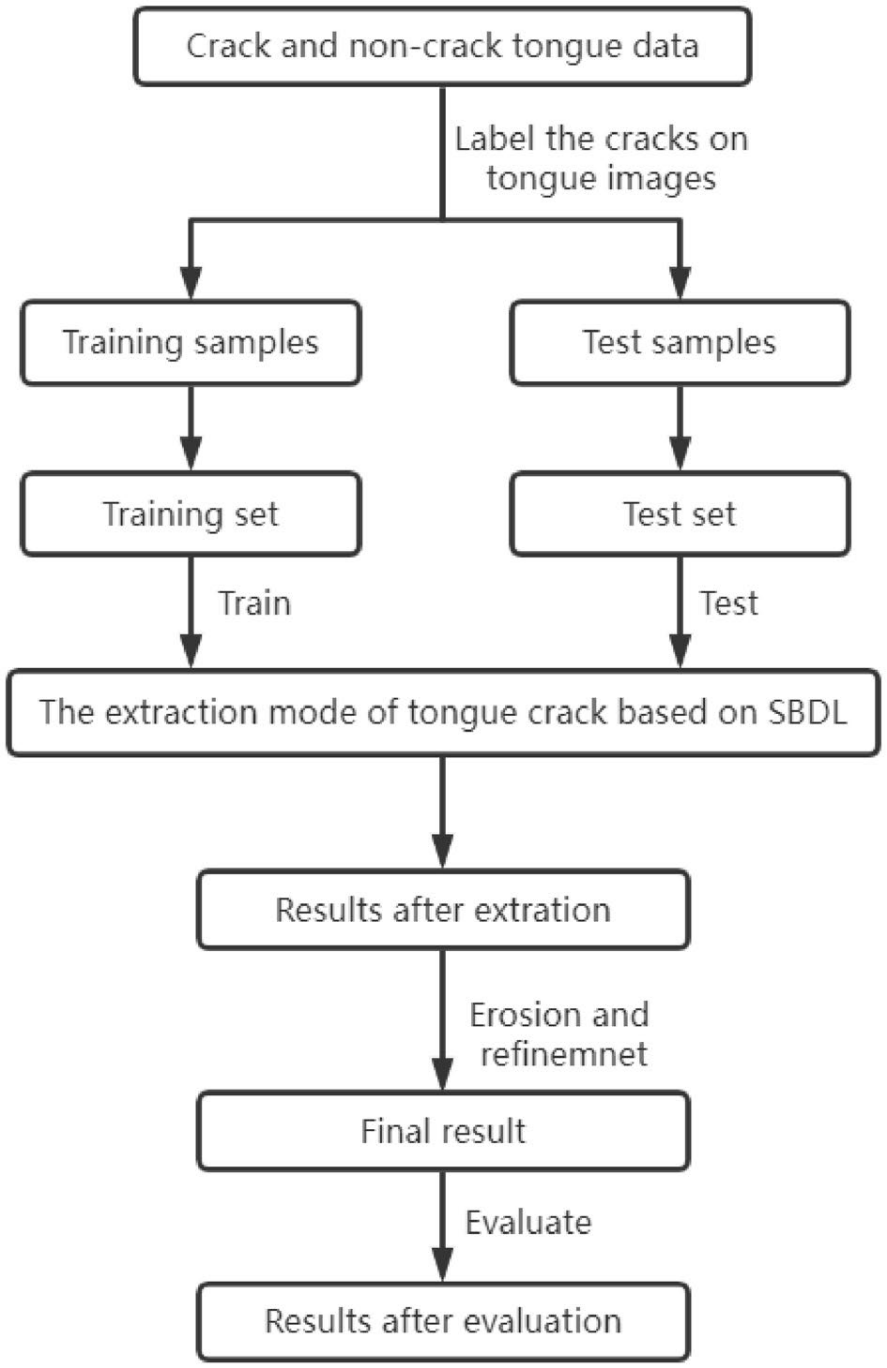
Flow chart of tongue crack extraction based on SBDL.

The flow chart of tongue crack extraction based on SBDL is shown in Fig. 2, which mainly includes five steps: tongue crack labeling, model training, model testing, optimization of tongue crack extraction results and result evaluation. Firstly, the data of cracked tongue and non-cracked tongue obtained by the tongue image segmentation model is allocated to training samples and test samples; secondly, the annotation tool Crack-Tongue is used to label the cracks in tongue image with the size of 400 × 400 to get the ground truth of tongue crack, and then put them into the corresponding training set and test set; thirdly, the tongue crack extraction model is trained by using the training set and SBDL network; fourthly, the test results with the size of 50 × 50 are optimized by means of the erosion and refinement operations to obtain the final results; finally, the results are evaluated quantitatively.

### Tongue crack image preprocessing and data enhancement

The tongue images used in this paper were provided by the Four Diagnostic Information Comprehensive Laboratory of Shanghai University of Traditional Chinese Medicine, including 176 images with tongue crack and 140 images without tongue crack. These tongue images are assigned to training samples and test samples in a ratio of 8:2, which means that there are 141 images with tongue crack and 112 images without tongue crack in training samples, and there are 35 images with tongue crack and 28 images without tongue crack in test samples. Since the tongue image dataset is too small, overfitting is easy to occur during training, which makes the training results more specific to a certain type of images and lacks sufficient generalization ability. Therefore, it is necessary to expand the number of tongue crack samples.

**Figure 3:**
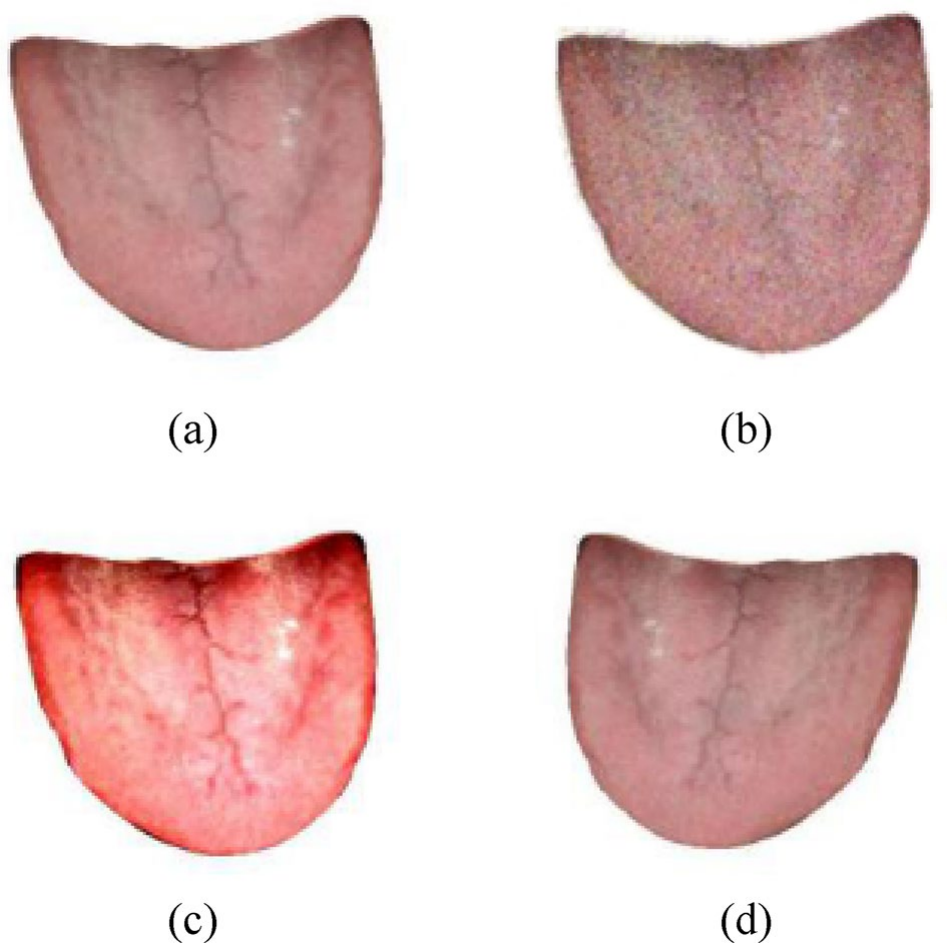
Data enhancement of tongue image. (**a**) Original tongue image. (**b**) Tongue image after adding salt and pepper noise. (**c**) Tongue image after changing contrast. (**d**)Tongue image after horizontally mirroring.

As shown in Fig. 3, the methods of data enhancement are as follows:

1. Adding salt and pepper noise. Salt and pepper noise, also known as impulse noise, is a common noise. After the image is subjected to this kind of noise, the gray value of the noise point is very different from the surrounding pixels. Usually, even if the image suffers very little salt and pepper noise, its details will be greatly destroyed^15^. Visually speaking, images subjected to salt and pepper noise will randomly produce small white or black dots.
2. Changing the contrast. There are many ways to change the contrast, which can be mainly divided into three types: image sharpening, smooth denoising and grayscale adjustment according to the processing purpose. This paper mainly uses grayscale adjustment to change the contrast.
3. Horizontal mirroring. Horizontal mirroring refers to mirror swapping the left and right parts of the image around the vertical center axis of the image.

After the above three image processing methods, the crack tongue data in training samples has been expanded from the original 141 to the existing 564. Therefore, there are 564 positive and 112 negative samples used as training samples; and there are 35 positive samples and 28 negative samples are used as test samples.

The model is trained by the SBDL network, the tongue crack image and the ground truth of tongue crack, so the tongue cracks in tongue crack image need to be labeled with the relevant labeling tools.

**Figure 4:**
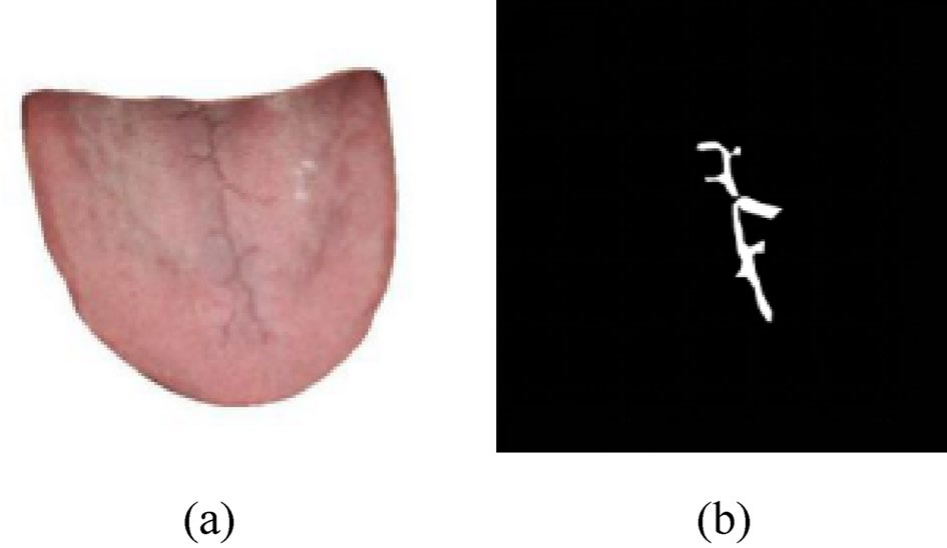
Tongue crack labeling effect. (**a**) Original image of cracked tongue. (**b**) Ground truth image of tongue crack.

The self-developed Crack-Tongue labeling tool is used to label tongue crack. The tool uses the shortest path between the labeling points to label based on the magnetic lock sleeve, and obtains a polygon that can fit the contour of the tongue crack. The labeling result is shown in Fig. 4.

### Construction of tongue crack extraction model based on SBDL

#### SBDL Network

Deep learning networks have two important factors that determine the speed of computation: the amount of training data and the computation amount of network parameters and floating-point numbers. Usually, in order to improve the accuracy of the training model, deep learning needs to use a large number of data samples to perform deeper feature learning under the adjustment of multiple network parameters. In order to improve the training speed and reduce the amount of calculation, the SBDL network divides the network into two stages: semantic segmentation stage and decision stage^14^. It uses the public crack detection to train and test, which achieves very good results. It uses a small data set to train a crack detection network model with small amount of calculation and high precision. Considering the surface crack detection problem as a binary image segmentation problem, pixel-level semantic segmentation can firstly be achieved through a semantic segmentation network, and then the segmentation results can be used as the input of the next decision stage.

The first is the segmentation network, which is mainly used to detect the tongue surface for cracks, generate a mask, and locate the location of the tongue crack. The segmentation network consists of 11 convolutional layers and 3 max-pooling layers. Each max pooling layer reduces the size of the output feature map by half. The convolutional layer consists of a BN layer and a nonlinear ReLU layer. The BN layer adjusts the output to the range of the standard normal distribution. The ReLU layer is the activation function layer used to adjust the output value. Both BN and ReLU layers have the effect of increasing the convergence speed. The method of dropout is not used in the network structure, because the weight-sharing convolutional layers have already provided sufficient regularization in the case of small training set. In order to capture small cracks in large-resolution tongue images, the network uses a 15 × 15 convolutional layer, which greatly increases the receptive field and uses a pooling layer instead of a convolutional layer to reduce the size of the feature map, thereby preserving feature information as much as possible.

**Figure 5:**
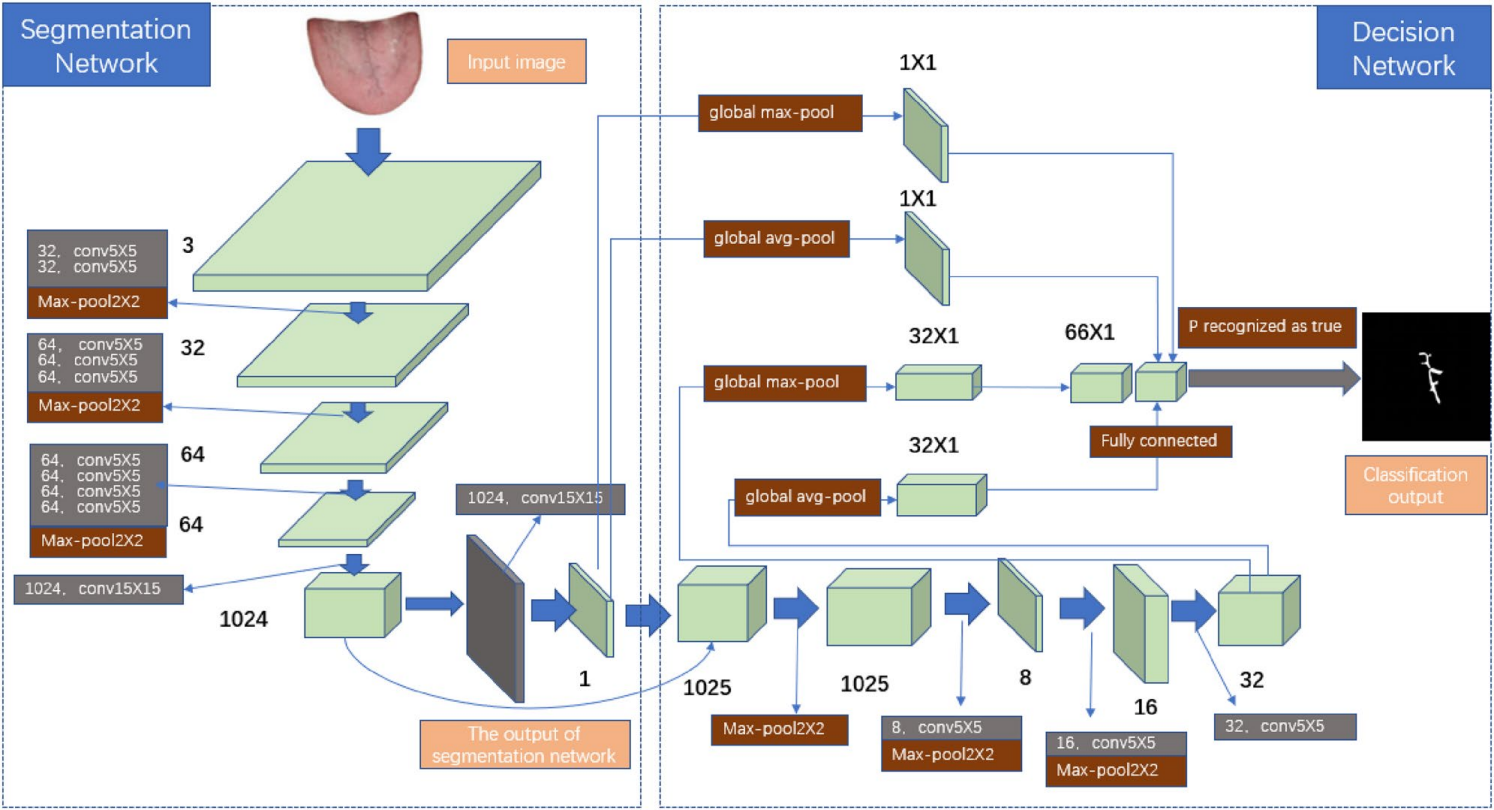
The network architecture of SBDL.

Then is the decision network which uses the output of segmentation network for the classification of cracked tongue images. Before entering decision network, the output of segmentation network will be superimposed with the last feature of the output layer, and both of them will be used as the input of decision network at the same time. This part of the network uses three 5 × 5 convolutional layers and three pooling layers as the method of convolution and downsampling, so that the network can capture the local shape of the image and observe a large range of shapes. After the last 5 × 5 convolution, a 32-channel feature is formed. Decision network also has a part of additional network, which performs the maximum and average global pooling of the output of the segmentation network. And it is attached to the final fully connected layer to prevent overfitting when the number of parameters is large. Finally, in order to simplify the network, different shortcut paths are added to the global maximum pooling layer and the convolutional layer, which is also one of the innovations of the SBDL network. The network architecture of SBDL is shown in Fig. 5.

#### Training of tongue crack extraction model

The tongue crack extraction model was trained by using the SBDL network in tensorflow 1.13 with CUDA10.0 and cuDNN7.5.0. The whole training process adopted stochastic gradient descent (SGD) to achieve a faster convergence speed. For the binary classification problem, the segmentation network only classified a single pixel in the image, used Mean Square Error (MSE) loss regression to perform bounding box regression, and used the cross-entropy loss function as a single pixel binary classification training strategy. The two networks were trained separately. Firstly, the segmentation network was trained, and then the decision network was trained while the weights of the segmentation network remained unchanged. The decision network only fine-tuned the weights during training, which largely avoid the risk of overfitting. Both stages were trained using SGD with a learning rate and cross-entropy loss rate of 0.005 and 0.1. Due to the image size and GPU memory constrained, the batch size was set to 2. Although the selection of training samples was random, in order to balance the number of cracked and non-cracked tongue images, the network detected cracked tongue image when the iteration number was even and non-cracked tongue image when the iteration number was odd. This approach ensured that the network detected tongue images at a constant rate. Otherwise, the imbalance of feature learning would bring poor generalization ability, overfitting and low learning speed to the model. The experiments are conducted on a 64-bit windows server with one Intel Core i7-10700 CPU, a 16G memory and one Nvidia RTX 3060 GPU.

### Evaluation indicators

The extraction of tongue cracks belongs to semantic segmentation. Semantic segmentation is pixel-level classification. The indicators used to quantitatively evaluate the effect of semantic segmentation are Mean Pixel Accuracy (MPA), Mean Intersection over Union (MIoU) and Frequency Weighted Intersection over Union (FWIoU). And Class Pixel Accuracy (CPA) and Intersection over Union (IoU) are specially added as the evaluate indicators of tongue crack segmentation. The formulas are as follows:

1 $$CPA = \frac{{\mathop \sum \nolimits_{i = 0}^{n} P_{ii} }}{{\mathop \sum \nolimits_{i = 0}^{n} \mathop \sum \nolimits_{j = 0}^{n} P_{ij} }}$$

2 $$MPA = \frac{1}{n + 1}\mathop \sum \limits_{i = 0}^{n} \frac{{P_{ii} }}{{\mathop \sum \nolimits_{j = 0}^{n} P_{ij} }}$$

3 $$IoU = \frac{{\mathop \sum \nolimits_{j = 0}^{n} P_{ii} }}{{\mathop \sum \nolimits_{i = 0}^{n} \mathop \sum \nolimits_{j = 0}^{n} P_{ij} + \mathop \sum \nolimits_{i = 0}^{n} \mathop \sum \nolimits_{j = 0}^{n} P_{ji} - P_{ii} }}$$

4 $$MIoU = \frac{1}{n + 1}\mathop \sum \limits_{i = 0}^{n} \frac{{P_{ii} }}{{\mathop \sum \nolimits_{j = 0}^{n} P_{ij} + \mathop \sum \nolimits_{j = 0}^{n} P_{ji} - P_{ii} }}$$

5 $$FWIoU = \frac{1}{{\mathop \sum \nolimits_{i = 0}^{n} \mathop \sum \nolimits_{j = 0}^{n} P_{ij} }}\mathop \sum \limits_{i = 0}^{n} \frac{{P_{ii} }}{{\mathop \sum \nolimits_{j = 0}^{n} P_{ij} + \mathop \sum \nolimits_{j = 0}^{n} P_{ji} - P_{ii} }}$$

In the formula, *n* is the number of all image categories except the image background;${P}_{ij}$ is the pixels number that class *i* is misclassified into class *j*, ${P}_{ji}$ is the pixels number that class *j* is misclassified into class *i,* ${P}_{ii}$ is the pixels number that class *i* is correctly classified into class *i*.

In addition to MIoU, general image processing algorithm performance evaluation adopts other three indicators: Sensitivity (SE), Specificity (SP) and Accuracy (ACC), which are defined as follows:

6 $$SE = \frac{TP}{{TP + FN}}$$

7 $$SP = \frac{TN}{{TN + FP}}$$

8 $$ACC = \frac{TP + TN}{{TP + FP + TN + FN}}$$

In the formula, TP means the positive samples are correctly predicted, and TN means the negative samples are correctly predicted; FP and FN mean the samples are mispredicted, the former means the positive samples are wrongly predicted as negative samples, and the latter means the negative samples are wrongly predicted as positive sample. SE refers to the number of positive samples correctly identified, and specificity (SP) refers to the number of negative samples correctly identified. Accuracy (ACC) refers to the total number of samples correctly identified.

At the same time, considering the application of tongue crack recognition in actual situations, this paper also adopts the professional evaluation for tongue cracks in traditional Chinese medicine (TCM). The experts of TCM usually use three terms: single crack, double cracks and multiple cracks to describe the number of tongue cracks, and use shallow cracks and deep cracks to describe the depth of tongue cracks. Therefore, in the objective study of tongue diagnosis in modern Chinese medicine, the visible index is commonly used to indicate the number of cracks, and the depth index indicates the depth of the crack. The specific formulas of the visible index and the depth index are as follows:

9 $$\begin{array}{*{20}c} {FCI = k_{v} \times \frac{{S_{t} }}{{S_{f} }}} \\ \end{array}$$

10 $$\begin{array}{*{20}c} {FDI = k_{d} \times \left( {\frac{{G_{f} }}{{G_{t} }} - 1} \right)} \\ \end{array}$$

In the formula, ${k}_{v}$ is the constant factor of the visible index, which is set to 10 here. ${S}_{t}$ is the area of the cracked area, ${S}_{f}$ is the area of the non-cracked area. The smaller the FCI value, the fewer cracks the tongue surface has. ${k}_{d}$ is the constant factor of the shade index, which is set to 2 here.${G}_{f}$ is the average gray level of the non-crack area, ${G}_{t}$ is the average gray level of the crack area. The smaller the FDI value is, the shallower the crack on the tongue surface is.

## Results

Since there are various types of tongue cracks which have different shapes, the ability of the model to identify cracks is particularly important. Therefore, the ability of the SBDL model to extract multiple tongue cracks must be considered. The trained SBDL network model is used to extract and identify the tongue cracks in the test set, and finally the test results are further processed through morphological processing such as corrosion and refinement to obtain tongue cracks.

In this paper, the extracted tongue crack image is corroded and refined with a rectangular template of size 3 × 3, and the skeleton pixels of the tongue crack are extracted to obtain a more accurate tongue crack area.

### Extraction results of tongue crack based on SBDL

**Figure 6:**
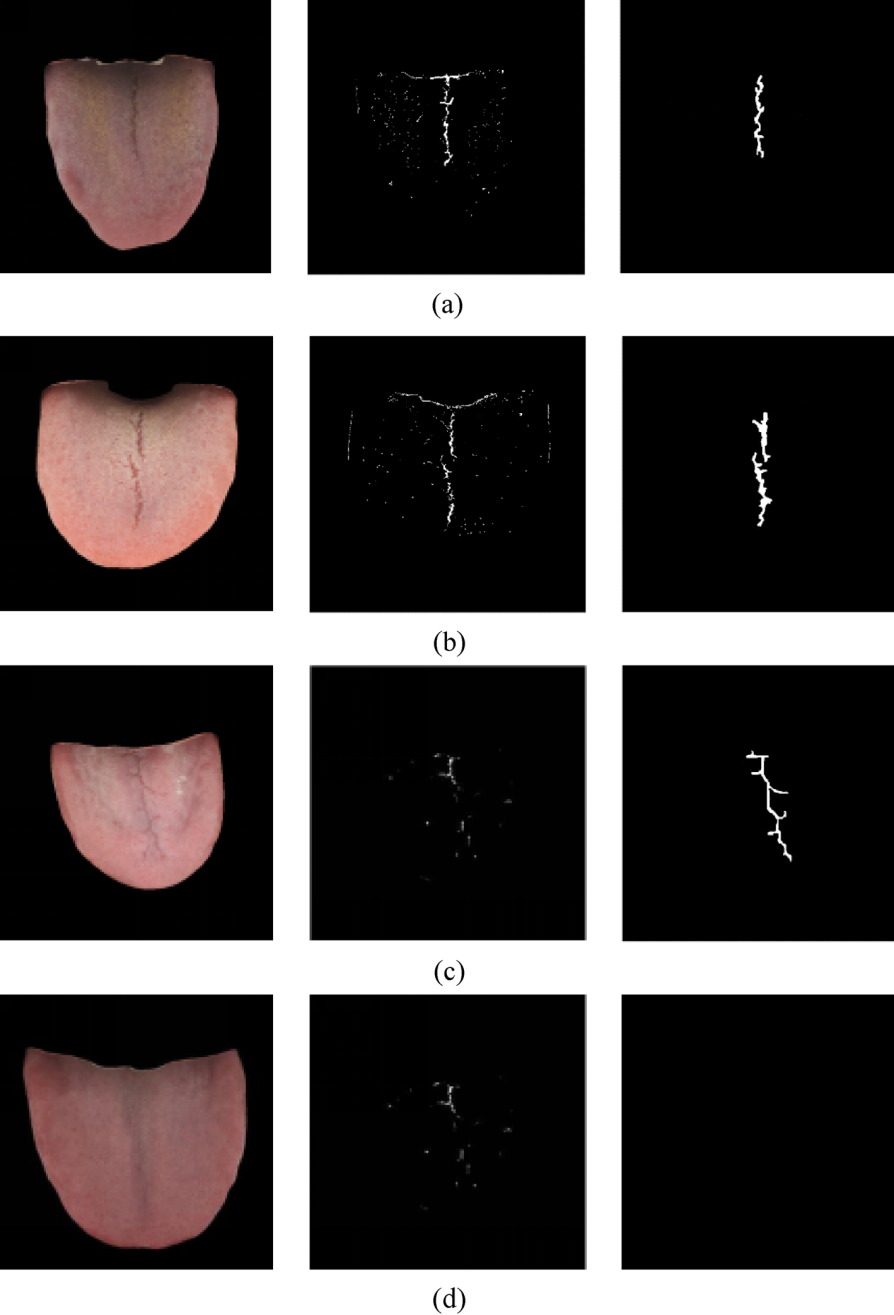
Comparison of crack extraction. (**a**) single-crack tongue (**b**) double-crack tongue (**c**) multi-crack tongue (**d**) non-crack tongue.

Generally speaking, when the color of the tongue crack area is not close to the surrounding tongue or tongue coating and the tongue crack edge is obvious, the crack extraction is easy to complete, and the effect is satisfactory. When the color difference between the two is large, even if the edge of the crack is not obvious, it can easily obtain better crack extraction results. However, when the tongue crack area is similar to the color of the surrounding tongue or tongue coating and the edge of the crack is not obvious, it is difficult to extract the tongue crack. But the model based on SBDL network can handle this situation well. In order to more intuitively illustrate the advantages of the SBDL network compared with the traditional tongue crack identification methods and further reflect the better robustness of the model, we compared it with the local grayscale threshold method, as shown in Fig. 6.

In Fig. 6, the left column is the original image of the cracked tongue, the middle column is the effect of extracting tongue cracks using the local grayscale threshold method, and the right column is the effect of using the SBDL model to extract tongue cracks.

### Model Evaluation

In order to more accurately measure the results of the crack extraction method based on SBDL network, the tongue crack extraction results are calculated according to formula (1)–(5) and compared with the crack extraction results based on Mask R-CNN, DeeplabV3+, U-Net, UNet++ and SegAN. The comparison results are shown in Table 1.

**Table 1:** Segmentation results of different models.

Model	MPA (%)	MIoU (%)	FWIoU (%)	FPS	Size (MB)
Mask R-CNN	99.7	62.6	99.5	3.36	244
DeeplabV3+	98.8	64.8	97.7	22.57	22.4
U-Net	99.2	73.1	98.4	20.8	94.9
UNet++	90.6	59.4	95.7	26.62	35
SegAN	98.2	62.1	98.1	80.42	596
SBDL	99.5	74.6	98.6	82.37	59.7

It can be seen from Table 1 that the MPA and the FWIoU of all six models are very close, which are higher than 90%, the MIoU and the FPS of the SBDL model reaches 74.6% and 82.37, which are higher than these indicators of the other five models, and the size of the SBDL model is 59.7 MB, which is smaller than this indicator of the other models except deeplabV3+ and UNet++.

It can be seen from Table 2 that the CPA and the IoU of tongue crack of the SBDL model reaches 67.1% and 50.1%, which are higher than these indicators of the other five models.

**Table 2:** Tongue crack segmentation indicators of different models($\overline{{\varvec{x}} }$, %).

Model	CPA	IoU
Mask R-CNN	36.9	25.4
DeeplabV3+	34.8	30.8
U-Net	48.6	40.7
UNet++	59.6	44.2
SegAN	14.9	7.4
SBDL	67.1	50.1

## Discussion

### Analysis of segmentation results based on SBDL

When the color of the tongue crack is slightly different from the surrounding tongue and the tongue crack isn’t single crack or double cracks, although the local gray threshold algorithm can also be used to obtain the approximate shape of the tongue crack, the complete crack area cannot be obtained, most of the crack area is missed. Not only that, tongue contours and tongue pricks are often misidentified as cracks and appear in the crack identification and extraction results. Therefore, the crack extraction results obtained by local grayscale threshold method not only miss some parts but also have fake cracks, the crack extraction effect is not good. However, the tongue crack extraction based on the SBDL network can obtain a more accurate tongue crack region. Furthermore, tongue pricks are not included in the crack results due to their different color and texture characteristics from the surrounding tongue tissue. On the contrary, the tongue contours and pricks can be better identified as non-cracks according to the color, texture and shape characteristics, so the crack extraction effect based on SBDL is satisfactory.

In addition, when identifying the non-crack tongue, the local grayscale threshold algorithm cannot exclude the color change of the tongue body well, so the area with the changing tongue color is misidentified as crack area, which misidentificates non-cracked tongue as cracked tongue. However, the SBDL model can better distinguish the difference between the tongue tissue with changing color and the tongue crack, so as to make a correct judgment, no cracks are mistakenly extracted from non-cracked tongue.

To sum up, the SBDL-based crack extraction method can better utilize the color, texture and shape features of the tongue to distinguish between the tongue crack and the tongue contours, prick, tongue body with changing color. Therefore, it can effectively solve the problem of inaccurate tongue crack extraction area caused by the closeness of the tongue crack to the surrounding tongue and tongue coating color and obtain better crack extraction results.

### Comparison of tongue crack segmentation results

**Figure 7:**
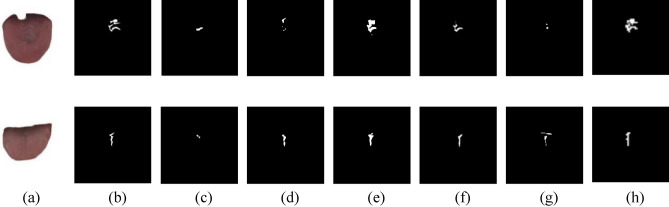
Segmentation results of different models. (**a**) Original images of tongue crack; (**b**) Ground truth images of tongue crack; (**c**) Segmentation results of Mask R-CNN^16^; (**d**) Segmentation results of DeeplabV3+^17^; (**e**) Segmentation results of U-Net^18^; (**f**) Segmentation results of UNet++^19^; (**g**) Segmentation results of SegAN^20^; (**h**) Segmentation results of SBDL.

Combined with the results of the Mask R-CNN, DeeplabV3+, U-Net, UNet++ and SegAN models shown in Fig. 7, a reasonable explanation can be obtained. It can be seen from Fig. 7 that the results obtained by the tongue crack extraction model based on Mask R-CNN are relatively rough, not only misidentifying the irrelevant areas around the tongue crack as cracks, but also ignoring the cracks that account for a relatively large proportion of the cracks. Both Mask R-CNN and SegAN can only roughly identify the location of the cracks, and the extracted cracks are incomplete. The segmentation results of tongue crack extracted by DeeplabV3+ are slightly better than Mask R-CNN and SegAN, and the extraction effect of single crack is better than that of multiple cracks, but the extraction of tongue crack details still needs to be improved. The tongue crack extraction effect of the U-Net is significantly better than that of Mask R-CNN, SegAN and DeeplabV3+, but the tongue crack width extracted by this model is significantly larger than the true value, and the accuracy of crack extraction is not high. The segmentation results of tongue crack by using UNet++ is more accurate than those using U-Net, but its segmentation result of multi-crack is incomplete.

It can be seen from Table 1 that the MPA and FWIoU of the six segmentation models are very close. The main reason is that the tongue crack area is very small, while background area is very big, so even if the tongue crack is not accurately segmented, the values of MPA and FWIoU will still be high. Therefore, the two indicators are difficult to reflect the true segmentation effect of tongue crack. Consequently, this paper specifically applied CPA and IoU as the evaluate indicators of the tongue crack segmentation performance. And the CPA and IoU of SBDL in Table 2 are significantly higher than the other five segmentation models, which means that the SBDL is better than other models in the segmentation performance of tongue crack.

In a word, the model based on SBDL can not only accurately identify the location of the tongue crack, but also can better extract the details of the tongue cracks, and the segmentation results of tongue crack are relatively complete, which are better than the other five models. And this also indicates that SBDL retains superior and stable performance when small number of training samples are available.

In order to further illustrate the ability of the SBDL model to judge the classification of tongue cracks, the classification ability of the SBDL model is evaluated by using formulas (6)–(8), and the results are shown in Table 3.

**Table 3:** The index of tongue-crack classification model based on SBDL network (%).

	Sensitivity	Specificity	Accuracy
SBDL	100	73.3	95.2

As can be seen from Table 3, the SBDL model has a high classification accuracy of 95.2%, and also has a satisfactory performance in sensitivity and specificity, reaching 100% and 73.3%, respectively. Therefore, this model has outstanding performance in the classification of tongue cracks.

The crack extraction model based on SBDL has a special network structure, which uses a combination of two different types of networks: a segmentation network using the pixel-level segmentation principle is responsible for generating a segmentation mask and determining the specific regional location of the tongue crack, and the decision network for binary classification is mainly responsible for judging whether the current tongue image has cracks. By using multiple convolutional and downsampling layers to ensure the extraction performance of complex shapes, the network is able to capture local shapes that span the entire image. The decision network takes full advantage of the output of the segment network and obtained features, reducing the use of a large number of feature maps. In the extraction and identification of tongue cracks, the decision to whether having cracks and the localization of cracks are equally important, and SBDL has satisfactory performance in both aspects. Because of the advantages that the structure of SBDL is combined by a segmentation network and a decision network, SBDL can achieve a good effect on tongue cracks dataset, and the performance improvement can also be generalized to other public datasets.

### Analysis of tongue crack indicators

The crack extraction model based on SBDL can obtain excellent results. In order to better apply it to the clinic, the four tongue crack extraction results in Fig. 6 are evaluated by using the visibility index (FCI) and the depth index (FDI), and the results are shown in Table 4.

**Table 4:** The results of tongue crack indicators.

Tongue crack indicator	Single-crack	Double-crack	Multi-crack	Non-crack
$FCI$	0.1775	0.4052	0.6264	0
$FDI$	0.0023	0.0196	0.0102	0

For the four tongue images of single crack, double-crack, multi-crack and none- crack in Fig. 7, the descriptive indicators of TCM can be calculated according to Formulas (9)–(10) by using the results obtained by the crack extraction model based on SBDL. As shown in Table 3, the FCI of single-crack, double-crack and multi-crack tongue images increases from low to high, and for non-cracked tongue image, FCI is 0, which shows that the SBDL model has high accuracy. The FDI of single-crack, multi-crack and double-crack tongues increases sequentially, and for non-crack tongue image, FDI is 0. The double-crack tongue image has the deepest crack, followed by multiple-crack, single-crack has the least crack, and non-crack tongue image has no cracks, so FDI is 0.

## Conclusion

In this paper, a tongue crack extraction and recognition method based on SBDL network are presented. There are five stages of the proposed method. Firstly, crack tongue and non-crack tongue images were assigned to training samples and test samples in a ratio of 8:2. Secondly, the labeling tool Crack-Tongue was used to label tongue cracks to obtain the ground truth of tongue crack, which were put into the corresponding training set and test set. Thirdly, the training set was used to train tongue crack extraction and recognition model. Fourthly, we input test set into the model to obtain test results. Finally, test results were processed by corrosion and refinement operations to obtain final crack extraction results. And the results of SBDL, Mask R-CNN, DeeplabV3+, U-Net, UNet++ and SegAN were compared by qualitative and quantitative evaluation methods, which verified the effectiveness of SBDL model. Although tongue crack images are different, the crack extraction model based on SBDL can achieve more accurate extraction and recognition for different tongue cracks.

### Ethical approval and consent to participate

The experimental protocol was established, according to the ethical guidelines of the Helsinki Declaration and was approved by the Human Ethics Committee of Shanghai Hospital of Traditional Chinese Medicine. Written informed consent was obtained from individual or guardian participants.

### Informed consent

Written informed consent for publication of this paper was obtained from the East China University of Science and Technology and Shanghai University of Traditional Chinese Medicine and all authors.

## Data Availability

The datasets generated and analyzed during the current study are not publicly available due to the confidentiality of the data, but are available from the corresponding author on reasonable request.
